# Differential Inhibition of LRRK2 in Parkinson's Disease Patient Blood by a G2019S Selective LRRK2 Inhibitor

**DOI:** 10.1002/mds.28490

**Published:** 2021-02-11

**Authors:** Jessica M. Bright, Holly J. Carlisle, Alyssa M. A. Toda, Molly Murphy, Tyler P. Molitor, Paul Wren, Kristin M. Andruska, Enchi Liu, Carrolee Barlow

**Affiliations:** ^1^ ESCAPE Bio, Inc. South San Francisco California USA; ^2^ Parkinson's Institute and Clinical Center Mountain View California USA

**Keywords:** pharmacodynamic, biomarker, Parkinson's disease, G2019S LRRK2, peripheral blood mononuclear cells

## Abstract

**Background:**

A common genetic mutation that causes Parkinson's disease (PD) is the G2019S *LRRK2* mutation. A precision medicine approach that selectively blocks only excess kinase activity of the mutant allele could yield a safe and effective treatment for G2019S *LRRK2* PD.

**Objective:**

To determine the activity of a G2019S mutant selective leucine‐rich repeat kinase 2 (LRRK2) kinase inhibitor as compared to a nonselective inhibitor in blood of subjects with genetic and idiopathic PD on two LRRK2 biomarkers, pSer935 LRRK2 and pThr73 Rab10.

**Methods:**

Blood was collected from 13 subjects with or without a G2019S *LRRK2* mutation with PD and one healthy control. Peripheral blood mononuclear cells were treated ex vivo with a novel G2019S LRRK2 inhibitor (EB‐42168) or the nonselective inhibitor MLi‐2. Quantitative western immunoblot analyses were performed.

**Results:**

EB‐42168 was 100 times more selective for G2019S LRRK2 when compared to wild‐type (WT) LRRK2. Concentrations that inhibited phosphorylation of pSer935 LRRK2 by 90% in homozygous G2019S *LRRK2* patients, inhibited pSer935 LRRK2 by 36% in heterozygous patients, and by only 5% in patients carrying only the WT allele. Similar selectivity was seen for pThr73 Rab10. MLi‐2 showed an equivalent level of inhibition across all genotypes.

**Conclusions:**

These findings demonstrate that EB‐42168, a G2019S LRRK2 selective inhibitor, lowers mutant G2019S LRRK2 phosphorylated biomarkers while simultaneously sparing WT LRRK2. Selective targeting of G2019S LRRK2 with a small molecule lays the foundation for a precision medicine treatment of G2019S *LRRK2* PD. © 2021 ESCAPE Bio, Inc. *Movement Disorders* published by Wiley Periodicals LLC on behalf of International Parkinson and Movement Disorder Society

Autosomal dominant Parkinson's disease (PD) is associated with missense mutations in the gene encoding the leucine‐rich repeat kinase 2 (LRRK2).^1^, ^2^ The most common disease causative (pathogenic) LRRK2 mutation is the Gly2019Ser (G2019S) *LRRK2* variant.^3^ All pathogenic LRRK2 variants identified to date, inclusive of G2019S, display abnormally high kinase activity as indicated by high autophosphorylation at the Ser1292 residue of LRRK2, implicating toxic gain of kinase activity as the cause of pathogenicity.^4^, ^5^, ^6^, ^7^ The recent identification of Rab GTPases as direct LRRK2 physiological substrates established an additional tool with which to demonstrate the increased kinase activity of the pathogenic LRRK2 variants. A subset of these Rab proteins show increased phosphorylation by pathogenic LRRK2 variants.^5^, ^8^

Pathogenic *G2019S* LRRK2‐induced increases in these established biomarkers provides a compelling biochemical link between the human genetics of the *G2109S* LRRK2 mutation and PD. This link underpins the active interest in therapeutic targeting of LRRK2 to dampen disease‐associated increases in LRRK2 kinase activity in order to slow disease progression in people with mutations in *LRRK2* as well as in idiopathic PD.^9^, ^10^

Though autophosphorylation of LRRK2 at Ser1292 is sensitive to LRRK2 inhibition, a peripheral blood clinical biomarker assay for monitoring the phosphorylation status of the Ser1292 residue has not been robustly established to date. Conversely, both the downstream substrate phosphorylation of Rab10 at Thr73 (pThr73 Rab10) and an established indirect LRRK2 inhibitor sensitive phosphorylation site at Ser935 (pSer935 LRRK2) have been utilized as target modulation biomarkers for therapeutic trials of LRRK2 kinase inhibitors.^11^, ^12^, ^13^

The aim of the present study was to compare the biomarker sensitivity of a novel, highly selective G2019S LRRK2 inhibitor (EB‐42168), shown to inhibit G2019S LRRK2 100‐fold more potently than wild‐type (WT) LRRK2 (Garofalo A., et al. 2020), with a nonselective LRRK2 inhibitor (MLi‐2).^14^ Inhibition of pSer935 LRRK2 and pThr73 Rab10 were compared in an ex vivo assay of human peripheral blood mononuclear cells (PBMCs) isolated from fresh blood samples from subjects who were homozygous, heterozygous, or noncarriers for the G2019S *LRRK2* variant.

## Methods

### Standard Protocol Approvals, Registrations, and Patient Consents

Study ECH‐10‐17 was approved by the institutional review board for the Parkinson's Institute and Clinical Center (PICC). Each patient gave written informed consent before enrollment.

### Subjects

The study aimed to enroll approximately 3 patients homozygous for the G2019S *LRRK2* mutation with a diagnosis of PD by a movement disorder specialist (MDS), 3 patients heterozygous for the G2019S mutation with a diagnosis of PD by a MDS, and 3 patients with a diagnosis of PD by a MDS without a G2019S *LRRK2* or a pathogenic *Glucocerebrosidase* (*GBA1*) mutation, at least 1 patient with a diagnosis of PD by a MDS and heterozygous for a pathogenic *GBA1* mutation of either the N370S type or a stop codon, and at least 1 male control 40–60 years of age without any known neurological disease. Subjects could not be part of an ongoing therapeutic intervention trial. Genotypes were based on data from the clinical record (each subject underwent genotyping at PICC to determine *LRRK2* and *GBA1* status using 23andMe, University of Indiana allele‐specific sequencing, Greenwood laboratory allele‐specific sequencing, and/or Centogene's next‐generation sequencing), three were then reconfirmed by sequencing during the study as described below.

### Patient Characteristics

Patient demographics and baseline characteristics are summarized in Table 1. Fourteen subjects were enrolled into the study (12 men and 2 women). A blood sample obtained from one donor (ESB‐01‐06) was not analyzable (due to hemolysis) and therefore not included in analysis or discussion. One subject was not affected by PD and served as a control. All patient genotypes except three were based on the clinical record as described above. DNA was sent for subjects ESB‐01‐02, ESB‐01‐03, and ESB‐01‐04 for confirmation of genotype using single‐nucleotide polymorphism (SNP) genotyping (rs34637584; dbSNP, Bethesda, MD) as described below (in section on Genotyping) and the results are shown in Table S1. Of the 13 subjects with analyzable samples, 1 subject was not affected by PD and served as a control (ESB‐01‐05), 3 subjects with PD were homozygous for the G2019S *LRRK2* mutation (ESB‐01‐04, ESB‐01‐11, ESB‐01‐14), 5 subjects with PD were heterozygous for the G2019S *LRRK2* mutation (ESB‐01‐02, ESB‐01‐03, ESB‐01‐08, ESB‐01‐12, ESB‐01‐13), including one also homozygous for a *GBA1* mutation (ESB‐01‐02), 4 subjects with PD did not have a mutation in the *LRRK2* gene (ESB‐01‐01, ESB‐01‐07, ESB‐01‐09, ESB‐01‐10), one of these four was heterozygous for a *GBA1* mutation (ESB‐01‐01), and finally 1 patient with PD was heterozygous for the Leu119Pro (L119P) *LRRK2* variant (ESB‐01‐10) (Table 1).

**TABLE 1:** Listing of subject diagnosis, sex, age, and genotype

Subject ID	Sex	Age (years)	PD diagnosis	G2019S *LRRK2* mutation	*GBA1* mutation
ESB‐01‐01	M	57	Yes	None	Heterozygous
ESB‐01‐02	M	70	Yes	Heterozygous	Homozygous
ESB‐01‐03	M	58	Yes	Heterozygous	None
ESB‐01‐04	F	70	Yes	Homozygous	None
ESB‐01‐05	M	55	No	None	None
ESB‐01‐07	M	74	Yes	None	None
ESB‐01‐08	M	82	Yes	Heterozygous	None
ESB‐01‐09	M	74	Yes	None	None
ESB‐01‐10	M	47	Yes	None^a^	None
ESB‐01‐11	M	62	Yes	Homozygous	None
ESB‐01‐12	F	59	Yes	Heterozygous	None
ESB‐01‐13	M	71	Yes	Heterozygous	None
ESB‐01‐14	M	68	Yes	Homozygous	None

PD, Parkinson's disease; M, male; F, female.

^a^ Heterozygous for the leucine‐rich repeat kinase 2 (LRRK2) Leu119Pro variant.

### Genotyping

For confirmation of genotype, DNA was sent for subjects ESB‐01‐02, ESB‐01‐03, ESB‐01‐04, and SNP genotyping (rs34637584; dbSNP) results are shown in Table S1. SNP genotyping was performed by GENEWIZ (South Plainfield, NJ). In brief, genomic DNA was extracted from pelleted PBMCs and the region surrounding rs34637584 was amplified by polymerase chain reaction (PCR) using the KAPABiosystems KAPA2G Fast HotStart PCR kit with the following primers AGGGACAAAGTGAGCACAGAA (forward) and CACAATGTGATAGACTCTGTTTTCC (reverse) and program 95°C/3 minutes, (95°C/10 minutes, 58°C/15 minutes, 72°C/15 minutes) ×5 cycles, (95°C/10 minutes, 55°C/15 minutes, 72°C/15 minutes) ×35 cycles, 72°C/2 minutes, 4°C/hold. After enzymatic purification, double‐strand sequencing was performed using BigDye Terminator Cycle Sequencing. Data analysis was performed by GENEWIZ with DNASTAR Lasergene12 software. The threshold for SNP detection was set to 10%.

### Sample Size

This was a pilot study and only a descriptive summary of the data is reported.

### LRRK2 Kinase Inhibitors

EB‐42168 is a tool compound discovered as part of a small molecule discovery program aimed at identifying LRRK2 kinase inhibitors that are selective for the pathogenic G2019S LRRK2 variant.^15^ MLi‐2 (synthesized at WuXi in Tianjing, China), included as a reference inhibitor, was shown previously to exhibit similar binding affinity for both WT and G2019S LRRK2.^14^ As expected, MLi‐2 inhibits WT LRRK2 approximately 1000‐fold more than EB‐42168.

### Blood Collection and PBMC Isolation

Patient blood samples were drawn at the PICC using BD Vacutainer K2‐EDTA 10 mL tubes, lavender closure (TigerMedical, Cat#TM84811) and transported to ESCAPE Bio at room temperature via same‐day courier. All 14 patients participating in study ECH‐10‐17 were given a unique designation Subject ID in the form, ESB‐01‐XX (−01 to −14). Each participant is referred to by this Subject ID. PBMCs were purified upon arrival from 20–60 mL human whole blood via density centrifugation using Lymphoprep (StemCell Technologies, Cat#07861). Lymphoprep (15 mL) was added into the SepMate tube (StemCell Technologies, Cat#85450, 50 mL capacity) to fill the bottom cavity. Whole blood (10 mL) was then diluted with an equal volume (10 mL) of phosphate‐buffered saline (PBS) containing 2% (by volume) fetal bovine serum (FBS) before being added into the top part of the SepMate tube. After centrifugation at 1200*g* for 10 minutes at room temperature, PBMCs were collected by swiftly pouring the Sep‐Mate tube contents into a new 50 mL Falcon tube. The sample was then washed by adding PBS containing 2% (by volume) FBS to a final volume of 50 mL. PBMCs were pelleted by centrifugation at 300*g* for 8 minutes. The supernatant was discarded by pouring, and the PBMC pellet was resuspended in 11.5 mL of RPMI1640 (ATCC, Cat#30–2001) containing 10% FBS (by volume). The PBMC suspension was aliquoted into 22 1.5 mL tubes of 500 μL each for ex vivo treatment with or without LRRK2 inhibitors. The remaining 0.5 mL of PBMCs was pelleted at 300*g* for 3 minutes, the supernatant was aspirated, and the cell pellet was snap‐frozen on dry ice and stored at −80°C for later genotyping if deemed necessary.

### Ex Vivo Treatment with LRRK2 Inhibitor

At this stage, purified PBMCs were treated ex vivo with EB‐42168, the G2019S selective LRRK2 inhibitor, or the nonselective LRRK2 inhibitor MLi‐2. PBMCs were treated for 60 minutes at 37°C with a total of 10 concentrations each of MLi‐2 and EB‐42168 or DMSO control. Following incubation, the cells were pelleted at 300*g* for 5 minutes, the supernatant was carefully removed by pipetting and each PBMC pellet was lysed by resuspending the cell pellet in 50 μL of ice cold lysis buffer for every 20 mL patient blood originally purified. Lysis buffer was made at 10× concentrate then diluted with 10 mL of water containing one cOmplete Protease Inhibitor Tablet (Sigma, Cat#11836170001) to final concentrations of 150 mM NaCl, 20 mM Tris pH 7.5, 1 mM EGTA, 1 mM EDTA, 1% TritonX‐100. Just before lysis, protease and phosphatase inhibitors were added to chilled lysis buffer, 1× HALT (Thermo Fisher Scientific Cat#78430), 1× PMSF (Cell Signaling Technologies, Cat#8553), 1× PhosphataseArrest I (G Biosciences, Cat#786–782), 1× PhosphataseArrest III (G Biosciences, Cat#786–452), Benzonase (Sigma, Cat#E8263), and 1 μM benzamidine (Sigma, Cat#63226). PBMC cell lysates were incubated on ice for 30 minutes and then clarified by centrifugation at 20,800*g* for 15 minutes at 4°C. Supernatant lysate was removed to new tubes, mixed with 3× Reducing SDS Loading Buffer (Cell Signaling Technologies, Cat#7722 containing 0.04 M DTT) and heated at 65°C for 10 minutes. The samples were snap‐frozen and stored at −80°C until analyzed; any insoluble pellet was discarded.

### Antibodies

For LRRK2 immunoblots, rabbit anti‐pSer935 LRRK2 antibody (clone UDD2 10(12), Abcam, Cat#ab133450) was multiplexed with mouse total LRRK2 antibody (clone N241A/34, Antibodies Inc., Cat#75–253). For Rab10 immunoblots, rabbit anti‐pThr73 Rab10 (clone MJF‐R21, Abcam, Cat#ab230261) was multiplexed with mouse total Rab10 antibody (clone 13B661, US Biologics, Cat#030583). All primary antibody incubations were done overnight at 4°C in 5% BSA‐TBS containing 0.1% Tween‐20 (5% BSA‐TBS‐T).

### Immunoblotting

Immunoblots for pSer935 LRRK2, total LRRK2, pThr73 Rab10, and total Rab10 were performed in the linear range of detection for each antibody. For LRRK2 immunoblots, 13 μL of sample was loaded for each treatment condition in duplicate onto NuPAGE 3–8% Tris‐Acetate Midi Gels (Thermo Fisher Scientific, Cat#WG1603BOX) and electrophoresed at 150 V in NuPAGE Tris‐Acetate SDS running buffer (Thermo Fisher Scientific, Cat#LA0041). For Rab10 immunoblots, 13 μL of sample for each treatment condition was loaded in duplicate onto NuPAGE 10% Bis‐Tris Midi Gel (Thermo Fisher Scientific, Cat#WG1203BOX) and electrophoresed at 200 V in NuPAGE MES SDS running buffer (Thermo Fisher Scientific, Cat#NP0002). Proteins were then transferred electrophoretically to nitrocellulose membranes (Thermo Fisher Scientific, Cat#PB3310) at 25 V for 10 minutes for LRRK2 protein and at 20 V for 5 minutes for Rab10 protein using iBlot (Thermo Fisher Scientific, Cat#IB21001). Membranes were blocked at room temperature for 1 hour in 5%BSA‐TBS and subsequently incubated with primary antibodies overnight at 4°C in 5%BSA‐TBS containing 0.1% Tween‐20 (5% BSA‐TBS‐T). Prior to secondary antibody incubation, membranes were washed three times with TBS containing 0.1% Tween‐20 (TBS‐T) for 10 minutes each wash. For detection, membranes were incubated for 1 hour at room temperature with Donkey anti‐Mouse IRDye 680RD (LiCOR, Cat#925–68,072) secondary antibody multiplexed with Donkey anti‐Rabbit IRDye 800CW (LiCor, Cat#925–32,213) diluted in 5% BSA‐TBS‐T (1:10,000 dilution). Membranes were washed again with TBS‐T three times for 10 minutes each to prepare for imaging. Protein bands on each immunoblot were visualized via near infrared fluorescent detection using the Odyssey CLx imaging system (LiCOR). Signals were quantified using Image Studio (LiCOR) analysis software.

### Data Analyses

**FIG. 1:**
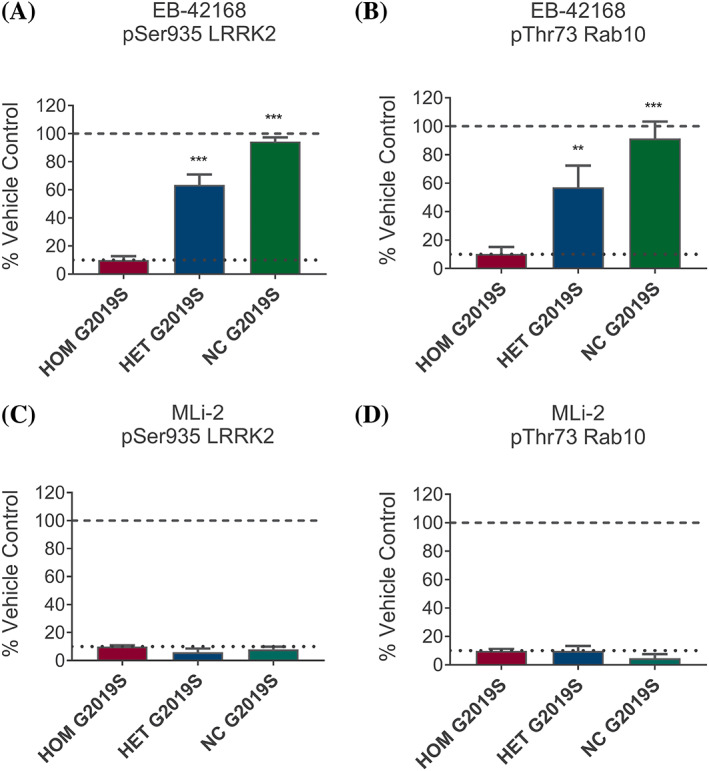
(**A**) The concentration of EB‐42168 that inhibited pSer935 LRRK2 by 90% in homozygous (HOM) G2019S *LRRK2* PBMCs (172 nM) resulted in significantly less inhibition of pSer935 leucine‐rich repeat kinase 2 (LRRK2) in heterozygous (HET) G2019S *LRRK2* PBMCs (36 ± 7.5%, *P* < 0.0001) and noncarriers (NC) peripheral blood mononuclear cells (PBMCs) (5 ± 3%, *P* < 0.0001). (**B**) The concentration of EB‐42168 that inhibited pThr73 Rab10 by 90% in HOM (582 nM) resulted in significantly less inhibition of pThr73 Rab10 in HET G2019S *LRRK2* (44 ± 15%, *P* < 0.0001) and NC PBMCs (9 ± 12%, *P* < 0.0001). (**C**) The IC_90_ concentration of MLi‐2 for pSer935 LRRK2 inhibition in HOM G2019S *LRRK2* PBMCs (10 nM) also inhibited the other genotypes by >90% (HET G2019S *LRRK2* = 95 ± 2.7%, *P* = 0.047; NC = 92 ± 2.2%). (**D**) The MLi‐2 IC_90_ concentration for pT73 Rab10 (33 nM) resulted in a similarly high level of inhibition in HET G2019S *LRRK2* (90 ± 3.6%) and NC (96 ± 2.9%). Bar height represents the average signal quantitated from immunoblots for pSer935 LRRK2 normalized to total LRRK2 relative to DMSO control (A and C) or pThr73 Rab10 normalized to total Rab10 relative to DMSO control (B and D). Error bars represent standard deviation (SD) of n = 3 HOM, 5 HET, and 5 NC of the G2019S *LRRK2* variant. **P < 0.005, ***P < 0.0001.

**FIG. 2:**
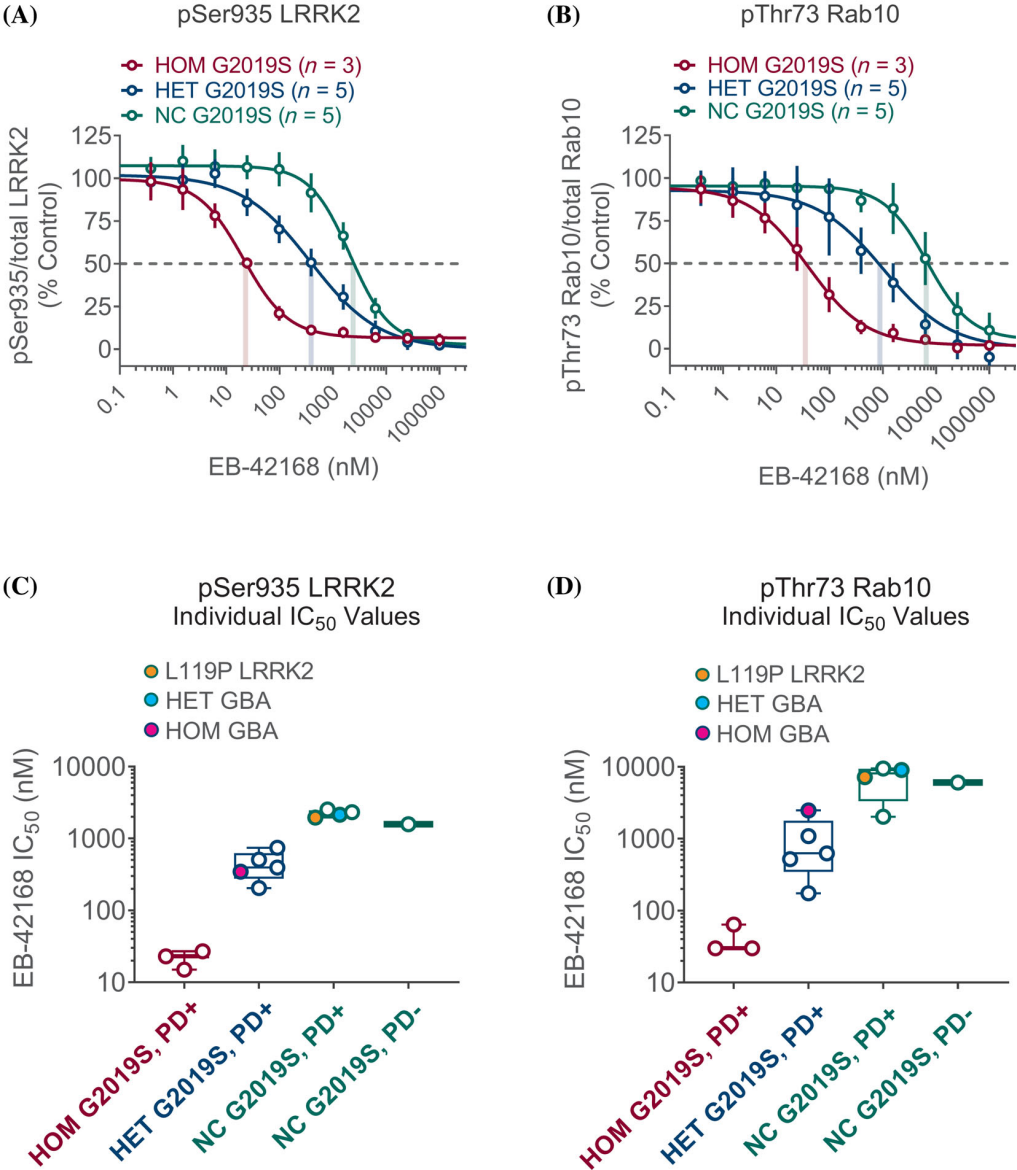
(**A**) EB‐42168 concentration response curves (range, 0.38 to 100 μM) for inhibition of pSer935 leucine‐rich repeat kinase 2 (LRRK2) are shown as the geometric mean for each genotype as indicated; correspondingly colored droplines show the aggregate IC_50_ values by genotype; error bars show geometric standard deviation (SD). (**B**) Similar representation for pThr73 Rab10. (**C**) Individual patient IC_50_ values for pSer935 LRRK2 ranged from 1579 to 2530 nM in G2019S *LRRK2* NC (open green circles, orange fill indicates presence of L119P *LRRK2* variant), 205 to 743 nM in heterozygous (HET) (open blue circles, turquoise and magenta fills indicate presence of additional HET and homozygous (HOM) *GBA1* variants, respectively), and 15 to 27 nM in HOM G2019S *LRRK2* (open red circles). (**D**) Individual patient IC_50_ values for pThr73 Rab10 ranged from 2022 to 9444 nM in G2019S *LRRK2* NC, 174 to 2480 nM in HET, and 30 to 64 nM in HOM G2019S *LRRK2* (colors of circles represent similar genotypes as in **C**).

**FIG. 3:**
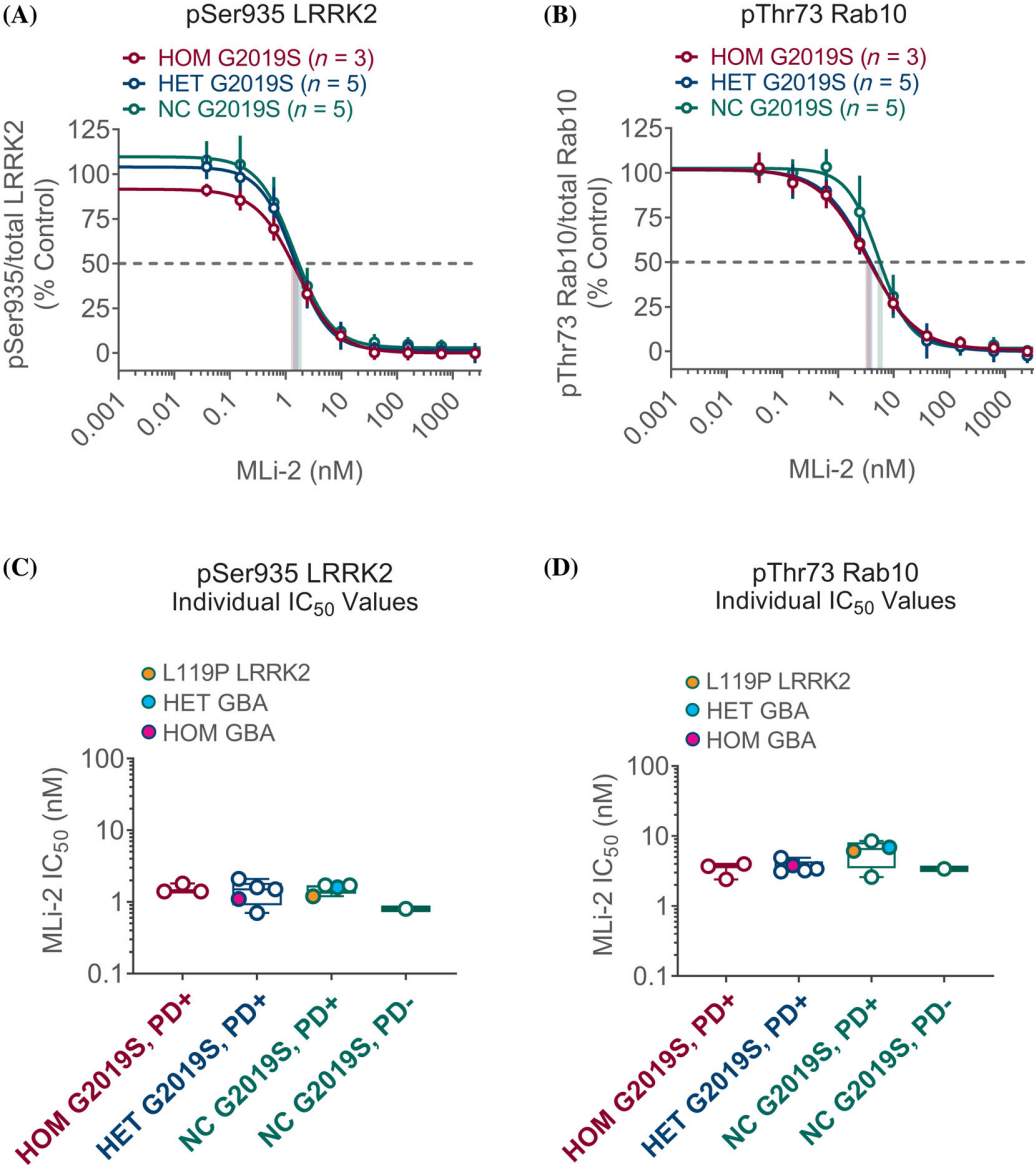
(**A**) MLi‐2 concentration response curves (range, 0.038 to 10 μM) for inhibition of pSer935 leucine‐rich repeat kinase 2 (LRRK2) are shown as the geometric mean for each genotype as indicated; correspondingly colored droplines show aggregate IC_50_ values by genotype; error bars show geometric standard deviation (SD). (**B**) Similar representation for pThr73 Rab10. (**C**) Individual patient IC_50_ values for pSer935 LRRK2 ranged from 0.8 to 1.7 nM in G2019S *LRRK2* noncarriers (NC) (open green circles, orange fill indicates presence of L119P *LRRK2* variant), 0.7 to 2.1 nM in heterozygous (HET) (open blue circles, turquoise and magenta fills indicate presence of additional HET and homozygous (HOM) *GBA1* variants, respectively), and 1.4 to 1.8 nM in HOM G2019S *LRRK2* (open red circles). (**D**) Individual patient IC_50_ values for pThr73 Rab10 ranged from 2.7 to 8.5 nM in G2019S *LRRK2* NC, 3.1 to 4.9 nM in HET, and 2.5 to 4.0 nM in HOM G2019S *LRRK2* (colors of circles represent similar genotypes as in **C**).

IC50 and IC90 values for both pSer935 LRRK2 and pThr73 Rab10 endpoints were calculated from quantified immunoblot data of full concentration response curves for each inhibitor in GraphPad Prism8 using a variable slope, four‐parameter logistic nonlinear regression model. This model assumes a single binding site to determine the concentration of each inhibitor that inhibited 50% (IC50) and 90% (IC90) of the biomarkers (pSer935 and pThr73) in PBMCs from each donor. Table 2 summarizes the inhibitor IC50 for each donor and the geometric means ± geometric standard deviations (SDs). Figure 1 is grouped by LRRK2 genotype and shows the percentage inhibition at IC90 for each compound calculated as the mean ± SD. A statistical comparison of the percentage inhibition at IC90 by genotype for each inhibitor was performed by ordinary one‐way ANOVA followed by a Dunnett's multiple comparison test using Prism8 software; statistically significant differences are represented: *P < 0.01, **P < 0.005, ***P < 0.0001. Figures 2A,B and 3A,B show IC50 concentrations grouped by LRRK2 genotype; the aggregate data are calculated as the geometric mean ± geometric SD. The full concentration response curves for each inhibitor, EB‐42168 (Fig. 2A,B) and MLi‐2 (Fig. 3A,B), are shown with subjects grouped by LRRK2 genotype, color‐coded droplines are used to show the aggregate IC50 for an inhibitor on the endpoint for that genotypes average. EB‐42168 in Figure 2C,D, and MLi‐2 in Figure 3C,D show each patient's individual IC50 value as a circle, grouped together by LRRK2 genotype in a boxplot, color‐filled circles highlight a patient with unique genetics within the broader genotype color‐coded groups of HOM G2019S (red), HET G2019S (blue), and NC G2019S (green). The IC50 and IC90 values reported for each donor were calculated from a single set of immunoblots for each marker with two technical replicates for each concentration of inhibitor.

**TABLE 2:** Summary of patient IC_50_ biomarker data for selective and non‐selective leucine‐rich repeat kinase 2 (LRRK2) inhibitors

G2019S*LRRK2*	PD status	*GBA1* variant	Subject ID ESB‐01‐	EB‐42168 (selective)				MLi‐2 (non‐selective)			
				pSer935 LRRK2		pThr73 Rab10		pSer935 LRRK2		pThr73 Rab10	
				IC_50_ (nM)^a^	Group IC_50_ (nM)	IC_50_ (nM)^a^	Group IC_50_ (nM)	IC_50_ (nM)^a^	Group IC_50_ (nM)	IC_50_ (nM)^a^	Group IC_50_ (nM)
HOM	PD+	NC	04	15	**21** ± 1.4	64	**39** ± 1.5	1.8	**1.5** ± 1.2	4.0	**3.3** ± 1.3
HOM	PD+	NC	11	27		30		1.4		3.7	
HOM	PD+	NC	14	23		30		1.4		2.4	
HET	PD+	HOM	02	345	**403** ± 1.6	2480	**686** ± 2.7	1.1	**1.3** ± 1.5	3.8	**3.6** ± 1.2
HET	PD+	NC	03	395		1088		1.6		4.9	
HET	PD+	NC	08	743		174		2.1		3.1	
HET	PD+	NC	12	205		624		0.7		3.4	
HET	PD+	NC	13	509		519		1.5		3.2	
NC	PD+	HET	01	2147	**2081** ± 1.2	9045	**5957** ± 1.9	1.6	**1.3** ± 1.4	8.5	**5.0** ± 1.6
NC	NKD	NC	05	1579		6045		0.8		3.4	
NC	PD+	NC	07	2530		9444		1.7		6.9	
NC	PD+	NC	09	2327		2022		1.7		2.6	
NC^b^	PD+	NC	10	1954		7184		1.2		6.1	

PD, Parkinson's disease; HOM, homozygous; HET, heterozygous; NC, noncarrier; PD+, confirmed Parkinson's disease; NKD, no known neurological disease.

^a^ IC_50_ values were calculated using a four‐parameter logistic nonlinear regression model which assumes a single binding site. Group IC_50_ values were presented in bold as the geometric mean ± geometric standard deviation.

^b^ Subject ESB‐01‐10 was negative for the G2019S *LRRK2* variant but positive for the L119P *LRRK2* variant.

## Results

### EB‐42168 Demonstrates Selective Inhibition in G2019S *LRRK2* Carriers with Limited Activity in G2019S *LRRK2* Noncarriers

Inhibition of LRRK2 was evaluated by immunoblotting for two different biomarkers: a phosphorylation site on LRRK2 (pSer935 LRRK2) that becomes dephosphorylated in response to inhibitor binding, and phosphorylation of the downstream LRRK2 physiological substrate, Rab10, at Thr73 (pThr73 Rab10). MLi‐2, which was shown previously to be equipotent in recombinant cell lines expressing WT LRRK2 or G2019S LRRK2, was used as a nonselective LRRK2 inhibitor control.

EB‐42168 inhibited pSer935 LRRK2 in homozygous G2019S carriers by 90% (IC_90_) at a concentration of 172 nM. At this concentration, inhibition of phosphorylation of Ser935 LRRK2 in heterozygous carriers was 36% and only 5% in noncarriers. (Fig. 1A). A similar pattern was seen for pThr73 Rab10, albeit higher concentrations were required to achieve IC_90_. The concentration of EB‐42168 (582 nM) that inhibited pThr73 Rab10 by 90% in homozygous G2019S PBMCs only inhibited phosphorylation of pThr73 Rab10 in heterozygous carriers and noncarriers by 44% and 9%, respectively (Fig. 1B). By contrast, approximately 10 nM MLi‐2 demonstrated similar IC_90_ inhibition across all genotypes for both biomarkers (Fig. 1C,D). No detectable change was noted in total LRRK2 or total Rab10 protein levels as a result of EB‐42168 or MLi‐2 exposure under these tested conditions (see Fig. S1 for an example immunoblot).

### Concentration Response Curves Confirm G2019S *LRRK2* Dependency of EB‐42168 in Human PBMCs and Lack Thereof for the Nonselective Inhibitor MLi‐2

Figure 2A,B show the full concentration response curves, grouped by *LRRK2* genotype, for the G2019S selective inhibitor EB‐42168 and the nonselective inhibitor MLi‐2. The aggregate IC_50_ values (illustrated by color‐coded droplines on the concentration response curves in Fig. 2A,B) for the selective inhibitor on pSer935 LRRK2 in homozygous G2019S *LRRK2* patients ranged from 15 to 27 nM compared with 1579 to 2530 nM in subjects carrying only the WT *LRRK2* allele, demonstrating a compound selectivity of approximately 100‐fold for the mutant G2019S *LRRK2* allele. A similar 100‐fold selectivity was seen for phosphorylation inhibition of the LRRK2 substrate Rab10 at Thr73, with an IC_50_ range of 30 to 64 nM in homozygous G2019S *LRRK2* carriers and 2022 to 9444 nM in subjects carrying only the WT *LRRK2* allele. The IC_50_ values for both biomarkers in the heterozygous G2019S *LRRK2* patients were intermediate between the IC_50_ values for homozygous G2019S LRRK2 patients and values in carriers of only the WT *LRRK2* allele, as shown in Table 2.

To demonstrate each individual subject's response to EB‐42168, box plots, grouped by *LRRK2* genotype, in Figure 2C,D show each subject's EB‐42168 IC_50_ for pSer935 and pThr73, respectively. Note that subject ESB‐01‐01 who carries a mutation in *GBA1* but is a noncarrier for the G2019S *LRRK2* mutation (turquoise filled circle, Fig. 2C,D), has a similar EB‐42168 IC_50_ value as subjects without a *GBA1* or a *LRRK2* mutation (shown generally in open green circles, Fig. 2C,D). This suggests that there is no interaction between EB‐42168‐dependent LRRK2 inhibition and *GBA1* status in PD. Consistent with this, patient ESB‐01‐02, homozygous for mutations in *GBA1* and heterozygous for the G2019S *LRRK2* mutation (magenta filled circle, Fig. 2C,D), had IC_50_ values similar to the four subjects heterozygous for the G2019S *LRRK2* mutation but who are noncarriers for *GBA1* mutations (shown generally in open blue circles, Fig. 2C,D). Interestingly, PD patient ESB‐01‐10, heterozygous for an alternate *LRRK2* variant (L119P), (orange filled circle, Fig. 2C,D) had an IC_50_ value similar to patients that did not carry the G2019S *LRRK2* mutation (shown generally in open green circles, Fig. 2C,D). This finding highlights the high selectivity of EB‐42168 for patients carrying the G2019S *LRRK2* mutation specifically rather than other *LRRK2* variants. Figure 3 uses the same box plot and color‐coded genetics to show that the nonselective LRRK2 inhibitor MLi‐2 inhibits all subjects equally on pSer935 LRRK2 and pThr73 Rab10, consistent with MLi‐2 lack of selectivity for G2019S when compared to WT LRRK2 protein. Unlike EB‐42168, full concentration response curve analyses highlighted a similarity in IC_50_ values for MLi‐2 for both biomarkers across all genotypes (Table 2).

## Discussion

This pilot study is the first to demonstrate that a G2019S LRRK2 selective agent, EB‐42168, reduced phosphorylation of both Ser935 LRKK2 and Thr73 Rab10 in human PBMCs in a gene dose‐dependent manner. At inhibitor concentrations that caused maximal reduction of phosphorylation of both biomarkers in samples from PD patients homozygous for the G2019S *LRRK2* mutation, only intermediate reduction was observed in samples from PD patients heterozygous for the G2019S *LRRK2* mutation, and little or no loss of phosphorylation was observed in the control subject and PD patients without the G2019S mutation. Of note, no loss of phosphorylation was observed in the PD patient with the L119P *LRRK2* mutation or in the PD patient without the G2019S mutation but heterozygous for a *GBA1* mutation. It would be interesting to determine the selectivity in subjects with other *LRRK2* variants, such as the G2385 variant, in the future. These data clearly demonstrate that a G2019S selective inhibitor can effectively reduce elevated biomarker activity associated with the pathogenic G2019S *LRRK2* mutation. EB‐42168 demonstrates 50% inhibition in G2019S heterozygous carriers, equivalent to nearly 100% inhibition in G2019S homozygous subjects. In contrast, the LRRK2 nonselective agent MLi‐2 showed similar activity regardless of genotype maintaining the same level of inhibition on mutant and WT LRRK2. These data evaluating the impact of a G2019S selective inhibitor in a system utilizing endogenous levels of LRRK2 in humans critically extends the findings from an artificial overexpression system.^15^

Though the therapeutic rationale for inhibiting pathogenic LRRK2 kinase activity is compelling, it is also complicated by concerns over on‐target safety liabilities associated with the loss of normal WT LRRK2 activity.^16^, ^17^ In a study evaluating three structurally distinct LRRK2 inhibitors, only partial inhibition of LRRK2 in the lung in macaque monkeys could be sustained without inducing on‐target pathological phenotypes in type II pneumocytes.^17^ Thus, the level of LRRK2 inhibition that can be safely achieved in the brain will likely be restricted by the need to preserve some level of normal LRRK2 function in peripheral tissues. Inhibition by 50% may not lead to any safety risk as suggested by data from human genetic studies showing no health consequences from loss of function of one *LRRK2* allele.^18^ This may be a limitation for nonselective inhibitors if greater than 50% inhibition is required for efficacy. By focusing only on subjects with one mutant G2019S *LRRK2* allele and using a selective inhibitor there is the potential to achieve efficacy while minimizing any safety risk.

Pathogenic variants in the *LRRK2* gene are one of the most common causes of familial autosomal dominant PD. The *LRRK2* G2019S variant is present in 4% of hereditary PD and up to 1% of apparently sporadic PD cases worldwide, making it among the most common genetic cause of PD.^19^ However, because the vast majority of *LRRK2* PD patients are heterozygous, carrying only one copy of the pathogenic allele, this patient population presents an excellent opportunity for a precision medicine approach to specifically target pathogenic kinase activity, while preserving the normal LRRK2 function of the healthy allele with a G2019S selective inhibitor.

Three prior studies have also used immunoblotting to quantify the phosphorylation of the LRRK2 biomarkers Ser935 LRRK2 and Thr73 Rab10.^20^, ^21^, ^22^ These studies demonstrate similar inhibition of biomarkers by nonselective LRRK2 inhibitors in PBMCs collected from idiopathic PD patients or controls. However, these immunoblotting approaches were unable to detect baseline changes in biomarker levels associated with disease or genotype. Efforts are underway to develop more sensitive, higher throughput, quantitative assays such as the ultrasensitive Quanterix SIMOA single molecular array assay, or high accuracy and sensitivity targeted mass spectrometry‐based assays, which could be deployed for future clinical trials.^11^, ^12^ A continued focus on clinical LRRK2 biomarker development^13^ may produce validated disease state‐dependent biomarker assays and/or pathogenic mutation sensitive biomarkers of elevated kinase activity. It would be interesting to test selective G2019S LRRK2 inhibitors in these emerging platforms which go beyond the ex vivo treatment evaluation of a G2019S LRRK2 selective inhibitor performed in this study in preparation for future clinical studies.

The current study sample size is small, with only one healthy control and descriptive summaries. However, one strength of this study is its range of different PD‐negative controls. No differences in reduction of phosphorylation between PBMCs isolated from idiopathic PD patients or controls were observed for the Ser935 LRKK2 and the Thr73 Rab10 sites in ex vivo studies using nonselective LRRK2 inhibitors.^20^, ^21^ The current ex vivo study demonstrates that the G2019S LRRK2 selective inhibitor EB‐42168 can differentially inhibit G2019S LRRK2 while sparing WT LRRK2. LRRK2 selective inhibitors offer the opportunity for a precision medicine proof of concept prospective clinical trials specifically in G2019S mutation carriers.

## Authors' Roles

K.A.: study supervision and coordination, review of manuscript. C.B.: study concept and design; study supervision; interpretation of data; drafting/revising manuscript for content. J.B.: study coordination and sample analyses; assay design; analysis and interpretation of data; drafting/revising manuscript for content. H.C.: scientific oversight; assay design; analysis and interpretation of data; drafting and revising manuscript for content. E.L.: study concept and design; study supervision and coordination; interpretation of data; authoring first draft of manuscript, revising manuscript. M.M.: identification and recruitment of subjects, oversight of IRB activities, study coordination; review of manuscript for content. T.M.: study coordination; advice on sample analysis and sample handling. A.T.: sample analyses; analysis and interpretation of data; drafting/revising manuscript for content. P.W.: review of data, review and edit of manuscript.

## Financial Disclosures of All Authors (for the Preceding 12 Months)

K.A. and M.M. were employees of PICC during the study conduct and report. They have no financial interests to disclose and no conflicts of interest. C.B., H.C., E.L., T.M., A.T., P.W., and J.B. are employees of ESCAPE Bio and own company stock. They have no other conflicts of interest.

## Supporting information


**Figure S1.** Representative immunoblots from subject ESB‐01‐12 are shown. Peripheral blood mononuclear cells (PBMCs) were treated ex vivo for 60 minutes with increasing concentrations of EB‐42168 (left), MLi‐2 (right), or DMSO control (lanes marked 0). Labels on the left correspond to pSer935 LRRK2 (pSer935), total LRRK2, pThr73 Rab10 (pThr73), and total Rab10.

**Table S1.** Sequencing results to confirm the *LRRK2* genotype of three subjects.
