# Supplementary material for: Differential Inhibition of LRRK2 in Parkinson's Disease Patient Blood by a G2019S Selective LRRK2 Inhibitor
Source: Mov Disord. 2021 Feb 11;36(6):1362–71. doi: 10.1002/mds.28490 (PMC8248170; doi:10.1002/mds.28490)
Supplement: Supplementary file 1 — Figure S1. Representative immunoblots from subject ESB‐01‐12 are shown. Peripheral blood mononuclear cells (PBMCs) were treated ex vivo for 60 minutes with increasing concentrations of EB‐42168 (left), MLi‐2 (right), or DMSO control (lanes marked 0). Labels on the left correspond to pSer935 LRRK2 (pSer935), total LRRK2, pThr73 Rab10 (pThr73), and total Rab10. [file MDS-36-1362-s002.zip › MDS_28490_ESCB PICC Clinical Study Manuscript Supplemental Figure 1.docx]

**Supplemental Figure 1 Legend.** Representative immunoblots from subject ESB-01-12 are shown. PBMCs were treated *ex vivo* for 60 mins with increasing concentrations of EB-42168 (left), MLi-2 (right), or DMSO control (lanes marked 0). Labels on the left correspond to pSer935 LRRK2 (pSer935), Total LRRK2, pThr73 Rab10 (pThr73) and Total Rab10.
