# Supplementary material for: Differential Inhibition of LRRK2 in Parkinson's Disease Patient Blood by a G2019S Selective LRRK2 Inhibitor
Source: Mov Disord. 2021 Feb 11;36(6):1362–71. doi: 10.1002/mds.28490 (PMC8248170; doi:10.1002/mds.28490)
Supplement: Supplementary file 2 — Table S1. Sequencing results to confirm the LRRK2 genotype of three subjects. [file MDS-36-1362-s001.docx]

**Supplemental Table 1:** Sequencing results to confirm the *LRRK2* genotype of three subjects.

| **Subject ID** | **Gene** | **SNP** | **Alleles** | **AA1** | **AA2** | **Mutation** |
| --- | --- | --- | --- | --- | --- | --- |
| ESB-01-02 | *LRRK2* | rs34637584 | G/A | G | S | c.g6055a p.G2019S |
| ESB-01-03 | *LRRK2* | rs34637584 | G/A | G | S | c.g6055a p.G2019S |
| ESB-01-04 | *LRRK2* | rs34637584 | A/A | S | S | c.g6055a p.G2019S |

c.g6055a, on cDNA, guanine (g) at 6055 is replaced by adenine (a)

p.G2019S, on protein, Glycine (G) at 2019 is replaced by Serine (S)

AA1: Allele 1 amino acid

AA2: Allele 2 amino acid
